# Air attenuation of high power XFEL beams

**DOI:** 10.1107/S1600577526004911

**Published:** 2026-06-05

**Authors:** Philip Heimann, Andrew Rosenstrom, Ming-Fu Lin, Razib Obaid, Taran Driver, James Cryan, Shanjie Xiao, Sena MacDonald, Sayed Rokni, Alyssa Prinz, Willy Langeveld, Lin Zhang

**Affiliations:** a Linac Coherent Light Source SLAC National Accelerator Laboratory 2575 Sand Hill Rd Menlo Park CA94025 USA; b Radiation Protection Department SLAC National Accelerator Laboratory 2575 Sand Hill Rd Menlo Park CA94085 USA; c Georgia Institute of Technology 770 State St NW Atlanta GA30332 USA; University College London, United Kingdom

**Keywords:** air, X-rays, absorption

## Abstract

The Linac Coherent Light Source II, a high-repetition-rate X-ray free-electron laser (FEL), produces high average power as well as high peak power. Air is a key radiation safety element, helping to contain the FEL beams from reaching accessible areas. At high average power, air absorption can produce a high-temperature and low-density channel along the X-ray beam path. In this case, the X-ray attenuation no longer follows the Beer–Lambert equation, exp(−μ*x*). Air attenuation measurements were performed with X-rays focused into a gas cell at the LCLS time-resolved AMO instrument. In the measurements, several parameters were varied: the repetition rate from 102 Hz to 33.2 kHz, the pulse energy from 18 to 340 µJ and the photon energy at 316 and 346 eV. The air transmission was observed to significantly increase with X-ray power. Simulations were also performed, which included the thermal processes related to the high average power. In comparison with the measurements, the calculated air transmission is higher, confirming that the simulations are conservative and suitable for radiation safety analyses.

## Abstract 2

The transmission of air, which is used to contain X-ray beams for radiation safety, is observed to significantly increase with X-ray power.

## 1. Introduction

Free-electron lasers (FELs) are high-brightness sources of X-rays that offer unique scientific opportunities as well as unique radiation protection challenges. The Linac Coherent Light Source II (LCLS-II) at SLAC presently produces X-ray pulses of a few 100 µJ at repetition rates up to 100 kHz, resulting in an X-ray beam power of the order of 10 W. In the future, the LCLS-II capability will increase with repetition rates up to 929 kHz, producing FEL X-ray beams up to several 100 W. At SLAC, one key radiation safety element is the presence of air, which helps to ensure that the FEL beam cannot reach areas accessible to personnel. However, as air attenuates the beam, it can be affected by the energy deposited by the beam. Specifically, the heating of the air as energy is absorbed from a FEL pulse creates a low-density channel for subsequent FEL pulses, a so-called tunneling effect. Tunneling has been observed with focused optical laser pulses (Cheng *et al.*, 2013 ▸). Low-density channels have also been experimentally measured using two bunches at 120 Hz at LCLS (Feng *et al.*, 2018 ▸), and 100-bunch trains at 10 Hz at EuXFEL. In Feng *et al.*, the two X-ray pulses were separated by 122.5 ns and the conditions were chosen to be similar to a gas attenuator, argon at 140 hPa in a tube with 165 mm length. One important difference between LCLS-II and EuXFEL is the evenly spaced pulse structure of LCLS-II as opposed to EuXFEL’s bunch trains.

This tunneling effect invalidates the standard Beer–Lambert exp(−μ*x*) calculations of the required air path length to ensure that the FEL beam does not reach personnel-accessible areas. A model, described in Section 4, has been developed in order to conservatively estimate the required air path length to aid in the design of radiation safety systems. The model includes heating of the air by the X-ray beam and the consequent reduction of the air density. The calculations do not include all processes, such as, for example, the ionization of the nitro­gen and oxygen molecules or the pulse structure of the X-rays. Feng *et al.* (2016 ▸) reported thermodynamic simulations of the X-ray attenuation by argon in a tube representative of a gas attenuator. In comparison with Feng *et al.*, the calculations presented here construct an accurate 3D temperature profile by using finite-element analysis (FEA). Yang *et al.* (2017 ▸) included hydro­dynamic motion in their simulations. In order to determine whether the modeling is reasonably conservative in the design of radiation safety systems, a plan for a series of measurements, referred to as ‘bootstrap tests’, has been developed in order to experimentally confirm that the radiation safety systems can mitigate hazards from the FEL beam. As a part of this bootstrap testing, measurements are performed at each step-wise increase of the beam power; this work documents the results of the air attenuation measurement at a power ramp-up step. The air attenuation measurements reported here were performed at the TMO (Time-resolved AMO) instrument (Walter *et al.*, 2022 ▸). These measurements and accompanying simulation results are part of the ramp-up of the LCLS-II accelerator.

## 2. Experimental setup

**Figure 1:**
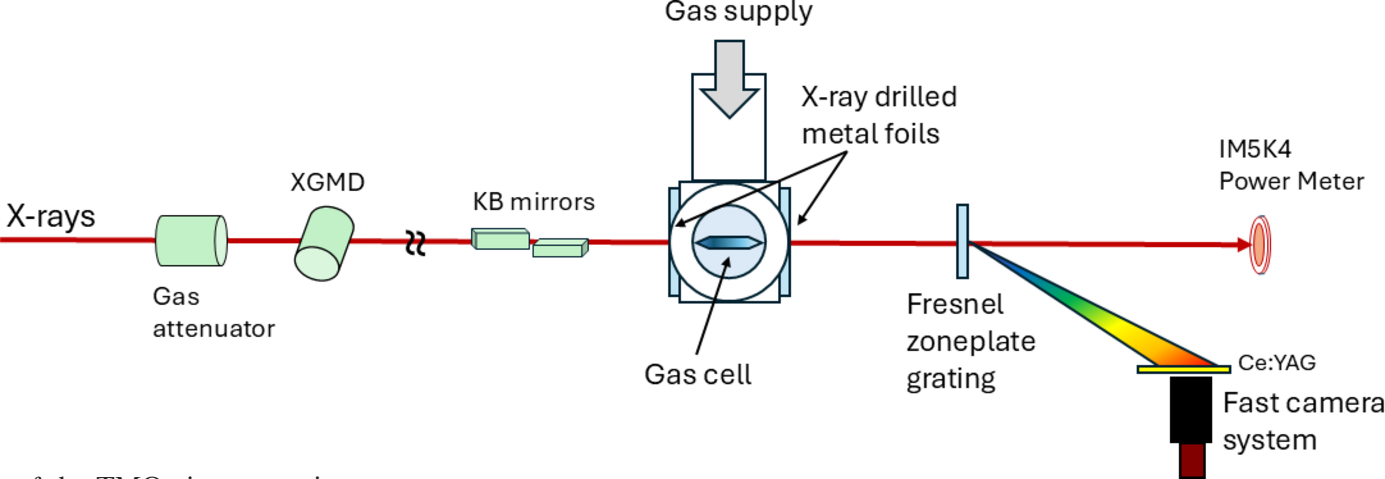
Schematic diagram of the TMO air attenuation setup.

The transmission of X-rays through the air in a gas cell is measured. A schematic of the experimental setup at TMO is shown in Fig. 1 ▸. The intensity of the X-rays from the undulators is monitored by an X-ray gas monitor detector (XGMD) and then focused by Kirkpatrick–Baez mirrors into interaction point 1 (IP1). A gas attenuator, AT1K0, provides variable attenuation. The air is contained in a differentially pumped gas cell with entrance and exit holes drilled by the X-ray beam. From measurements of pinhole dimensions after previous gas cell experiments and the calculated X-ray dose of 1600 eV per atom far above the steel damage threshold, it is considered that the pinhole diameters are significantly larger than the full width at half-maximum (FWHM) of the X-ray beam and that aperturing of the beam by the pinholes will be a small effect. The air is introduced using a tube at the top of the cell and is maintained at 1 atm by a pressure controller. The X-ray intensity downstream of the gas cell is observed by either a power meter (IM5K4) or an off-axis zone plate spectrometer. The power meter is capable of detecting high power, nominally up to 200 W, but has a slow response time of 1 s, and has a minimum sensitivity of 10 mW. The zone plate spectrometer can be damaged at powers above 1 W but can detect individual X-ray pulses at the repetition rate of 33 kHz. The measurements are performed in two steps. With the gas cell under vacuum, the X-ray power is measured by the power meter or zone plate spectrometer. The gas cell is then filled with air at 1 atm and the X-ray power is measured again. The transmission is calculated from the ratio of the two results, after normalizing using the XGMD pulse energy measurements to account for variations in the incident X-ray beam intensity. Power-dependent effects in the XGMD have not been observed at the LCLS. Calculations for the XGMD predict that, in the presence of the applied electric field, ions are pulled out of the X-ray beam path within 100 ns (K. Tiedtke, private communication).

The measurement parameters are listed in Table 1 ▸. The primary photon energy was 346 eV. At this energy, the low-power air absorption length is 3 mm. This is comparable with the gas cell length of 6.6 mm. Other measurements were performed at 316 eV. Compared with the pulse energy measured by the XGMD, the pulse energy at the gas cell is reduced by absorption in the three mirrors directing the beam to IP1. This beamline transmission was measured by the power meter and ranged from 0.71 to 0.73 at the two photon energies.

The X-ray power was changed mainly by varying the repetition rate and in some cases using the gas attenuator. After adjusting the bending of the KB mirrors, a focus of 2 µm (FWHM) was measured by the PF1K4 wavefront sensor downstream of the gas cell chamber. From the divergence of the X-ray beam, a beam width of 10 µm (FWHM) was calculated at the front and back of the gas cell. The photon energy was calibrated by observing the nitro­gen 1*s*–π* resonance at 401 eV (King *et al.*, 1977 ▸) with the zone plate spectrometer, at an air pressure of 7 torr in the gas cell. The calibrated photon energy differed from the nominal FEL energy by −4 eV.

**Figure 2:**
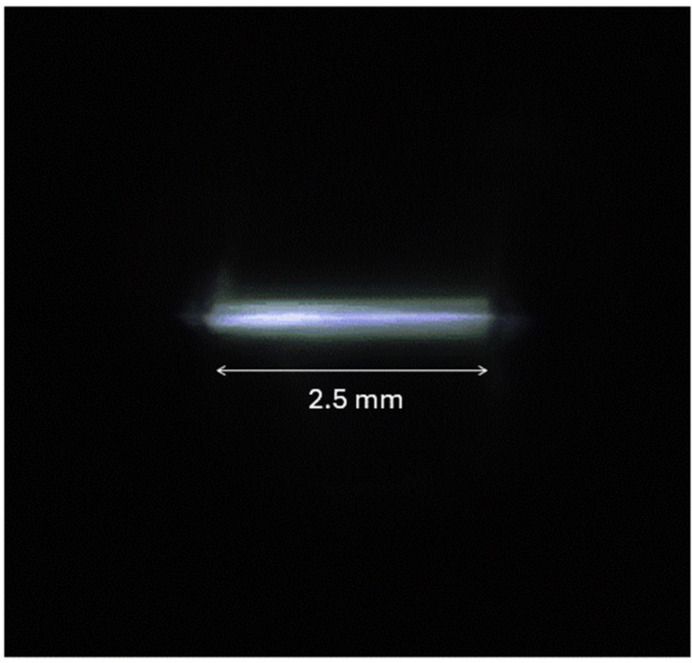
The nitrogen fluorescence from the gas cell at 346 eV photon energy. An accompanying video can be found in the supporting information.

There is a window on the side of the gas cell as well as windows on the differential pumping enclosure and on the chamber wall. A camera was positioned to view the nitro­gen fluorescence from the X-ray beam passing through the air. In Fig. 2 ▸, the repetition rate is 16.6 kHz, the photon energy is 346 eV and the average pulse energy is 340 µJ. The length of the observed emission is limited by the width of the cell window. The camera had an exposure time of 10 ms, which implies that 166 X-ray pulses are integrated over in each frame. The emission does show significant fluctuations. It is suggested that the fluctuations are caused in part by the LCLS source and in part by variations in the air absorption. LCLS pulse energy variations are observed pulse-by-pulse in the XGMD, σ = 11–12% (standard deviation/mean). The absorption variations can be seen in Video S1 of the supporting information by the shifting maximum fluorescence along the beam propagation direction, *z*. In addition to the average transmission, the peak transmission is relevant to FEL radiation safety.

## 3. Results

**Figure 3:**
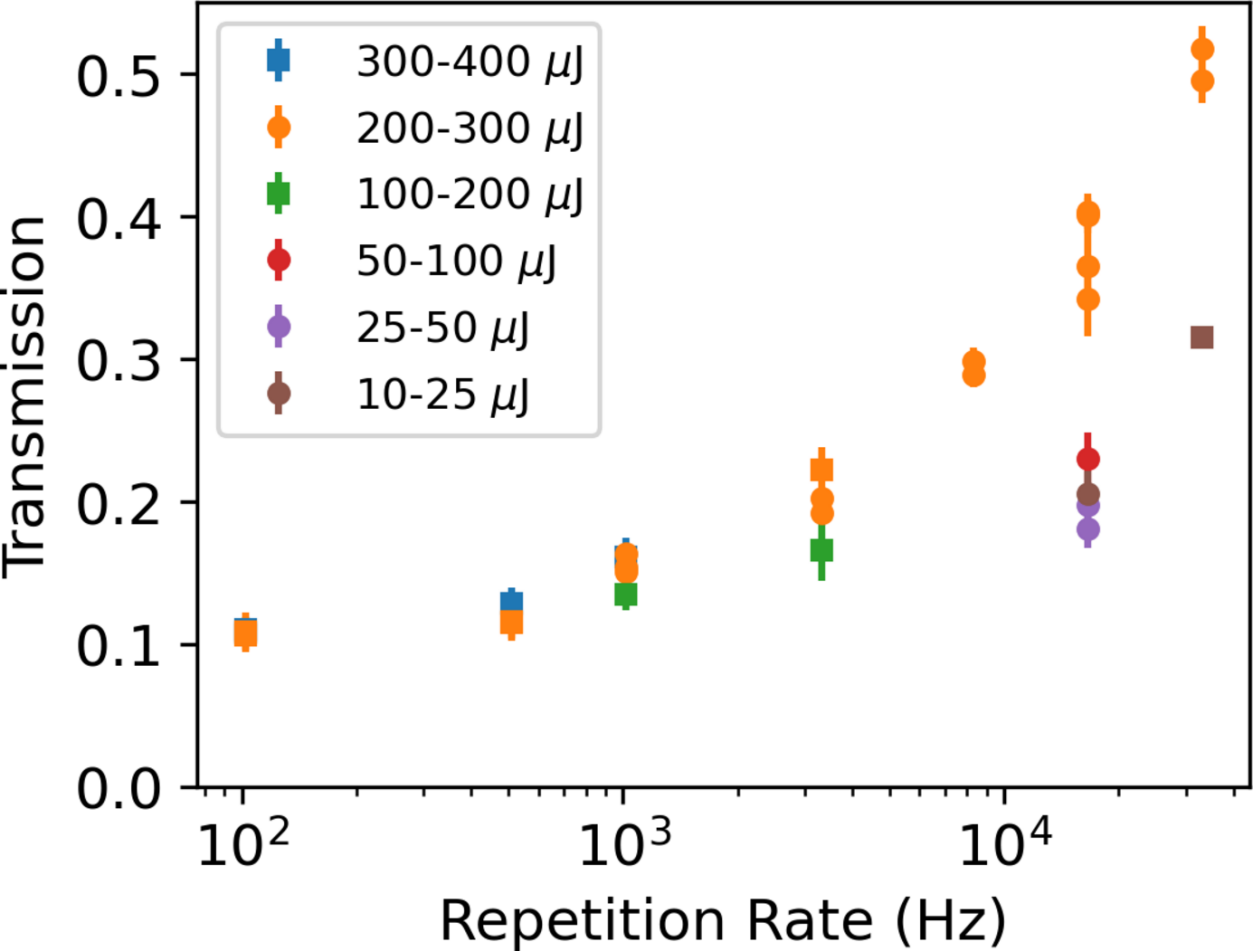
The air transmission at different repetition rates and pulse energies and 346 eV photon energy. The squares represent zone plate spectrometer and the circles power meter measurements.

Fig. 3 ▸ shows the X-ray transmission through the gas cell at 346 eV as a function of X-ray beam power. The transmission (*T*) is calculated according to
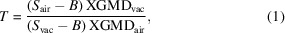
where *S*_air_ and *S*_vac_ are the average zone plate spectrometer or power meter signals with the gas cell filled with air and under vacuum, *B* the background and XGMD_air_ and XGMD_vac_ the average XGMD pulse energy with the gas cell filled with air and under vacuum. For the zone plate spectrometer, the background *B* was derived from a region of interest separated from the signal. For the power meter, background runs were performed without X-rays to determine *B*. In the data analysis, filters were applied to exclude events for which the X-ray beam was off. At lower powers the zone plate spectrometer was used and at high powers the power meter was used. For measurements with the zone plate spectrometer (squares in Fig. 3 ▸), the repetition rate was varied from 100 Hz to 3.3 kHz with the gas attenuator transmission set to 1, and in a second data set the repetition rate and gas attenuator transmission were both adjusted keeping the power at ∼0.8 W. For measurements with the power meter (circles in Fig. 3 ▸), the repetition rate was varied from 1 to 33.2 kHz with a gas attenuator transmission of 1, and at a fixed 16.6 kHz repetition rate the gas attenuator transmission was adjusted between 0.07 and 1. At low power, the transmission agrees with the room-temperature value of 0.11 calculated from the Center for X-ray Optics website (https://henke.lbl.gov/optical_constants/). At the higher pulse energies, the transmission follows a curve increasing with rising repetition rate. At each repetition rate, lower pulse energies reduce the transmission toward the room temperature value. Overall, the air transmission increases as the repetition rate and pulse energy become higher, showing the effect of a low-density channel.

**Figure 4:**
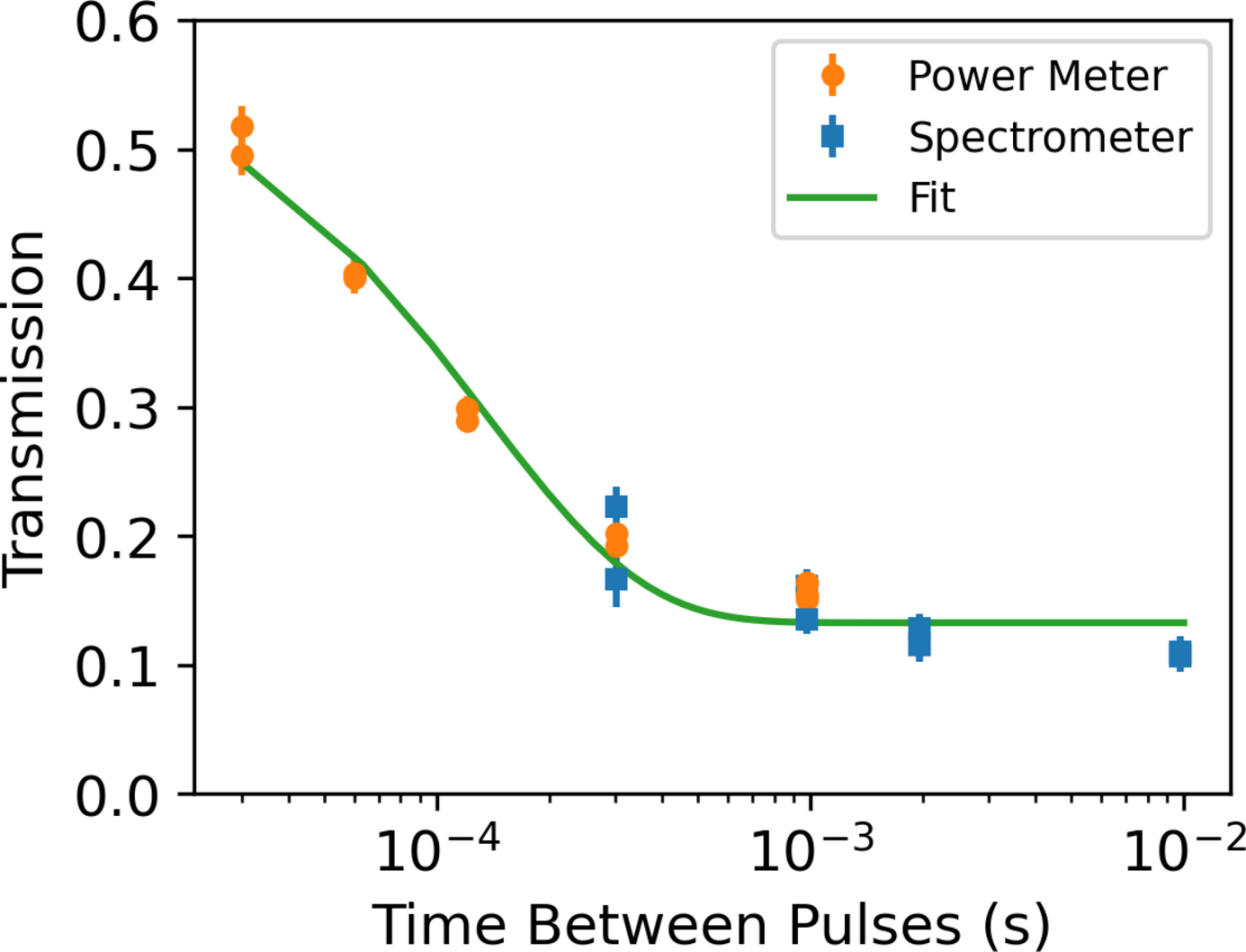
The air transmission as a function of the time between X-ray pulses at 346 eV photon energy.

To isolate the effect of repetition rate, data at similar pulse energies can be selected. Fig. 4 ▸ shows power meter and zone plate spectrometer measurements at 346 eV photon energy (orange and blue data points in Fig. 4 ▸) plotted with the time between X-ray pulses. Here, for the power meter measurements, the average pulse energies were between 0.20 and 0.26 mJ, and for the zone plate spectrometer measurements the average pulse energies were between 0.17 and 0.34 mJ. The curve shows a fit to the exponential function *c*_0_exp(−*t*/*t*_c_) + *c*_1_, with *t*_c_ evaluated to be 133 ± 15 µs. This time constant is interpreted as being related to the time for the low-density channel to be refilled by diffusion of the air molecules. It is noted that the power is also changing in Fig. 4 ▸. Cheng *et al.* (2013 ▸) described measurements and simulations of the gas dynamics for focused 40 fs optical laser pulses in 1 atm air and nitro­gen. In air, the channel gas density recovered with*t*_1/e_ ≃ 400 µs.

**Figure 5:**
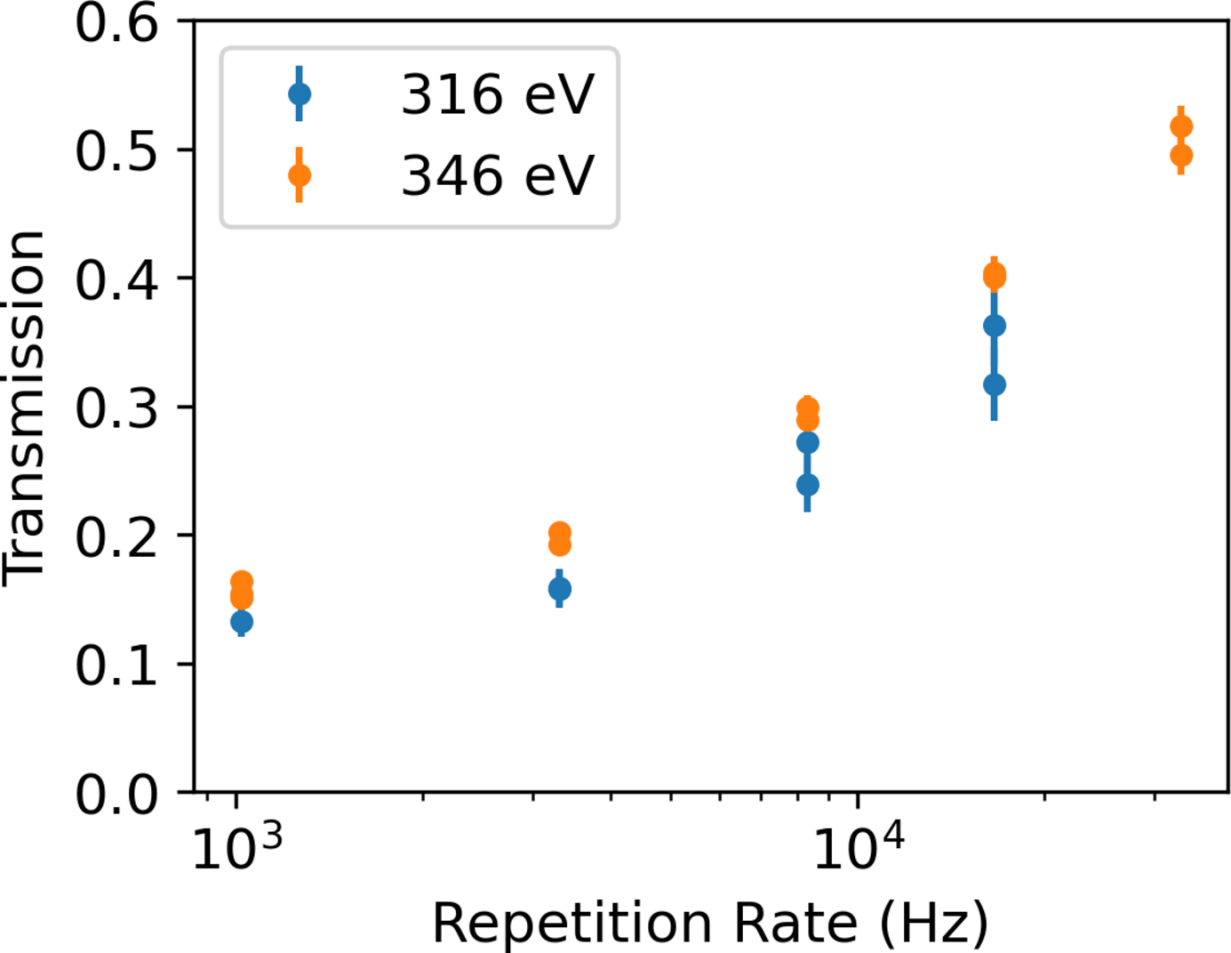
The air transmission at 316 eV photon energy, and at 346 eV for comparison.

Fig. 5 ▸ shows measurements performed at an additional photon energy, 316 eV, with the power meter. The repetition rate was varied from 1 to 16.6 kHz. For comparison, measurements at 346 eV photon energy with varying repetition rate are included. At 316 eV, the room-temperature calculated transmission is 0.06 (https://henke.lbl.gov/optical_constants/). As expected from the lower room temperature transmission, the transmission at 316 eV is similar and somewhat lower than that at 346 eV.

The measurement uncertainty is estimated as follows. For the power meter, two runs were recorded for each set of parameters, and the uncertainty, σ_PM_, was calculated as
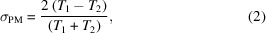
where *T*_1_ and *T*_2_ are the transmissions for the two runs. This analysis resulted in a transmission uncertainty of between 0.5 and 3%. For the zone plate spectrometer, the data from each run were divided into three parts and the uncertainty, σ_SP_, calculated according to
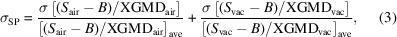
where, on the right side, σ[…] is the standard deviation of the three segmented data sets and […]_ave_ the average. This method resulted in an estimated transmission uncertainty of between 1 and 2%.

## 4. Simulations

### 4.1. General description and finite element model

The power absorption by a gas is more complicated to estimate than absorption by a solid. When an intense X-ray beam interacts with a gas, it can provoke a series of physics phenomena: photoemission, cascade photon emission, ionization and plasma creation. The time scale of these physics processes is below 1 ns. From then on, the heat transfer process in the gas is dominant, which leads to a temperature distribution. When the gas heats up, the temperature of the gas increases, the density of the gas on the beam path decreases, and the X-ray beam transmission increases. This is the so-called ‘tunneling’ effect (Feng *et al.*, 2016 ▸). The power absorption calculated using the exponential law *P*_abs_ = *P*_0_[1 − exp(−μ*t*)], where μ is the absorption coefficient and *t* is the thickness of the gas, is higher than the actual absorbed power. Ortega (2017 ▸) used a Monte Carlo method to simulate the physics of argon in a 50 mm tube at X-ray powers up to 700 W, and found that the simulated power absorbed by the gas is about 30% lower than the measured results using a calorimeter. This suggests that about 30% of 700 W X-ray power is dissipated by plasma, photon electrons, secondary photon emission, *etc.*

By neglecting the complex physics processes during the first nanosecond after the FEL beam interacts with the gas, we can focus on the thermal processes primarily related to the average power. We assume all the FEL power absorbed by the gas contributes to this thermal process. The temperature of the gas on the X-ray beam path increases, heat transfer occurs within the gas and eventually with the cell walls in contact with the gas. Finally, an equilibrium of the gas temperature and density distributions is reached.

In general, there are three heat transfer modes involved in the gas:

(i) Thermal conduction: this is the main heat transfer mode.

(ii) Convection: the contribution in heat transfer by natural convection of the gas can reduce the temperature of the gas by 30% (Zhang, 2022 ▸). By neglecting natural convection heat transfer, the beam power transmission is overestimated.

(iii) Thermal radiation: air in the infrared wavelength region is transparent, therefore thermal radiation is negligible.

**Figure 6:**
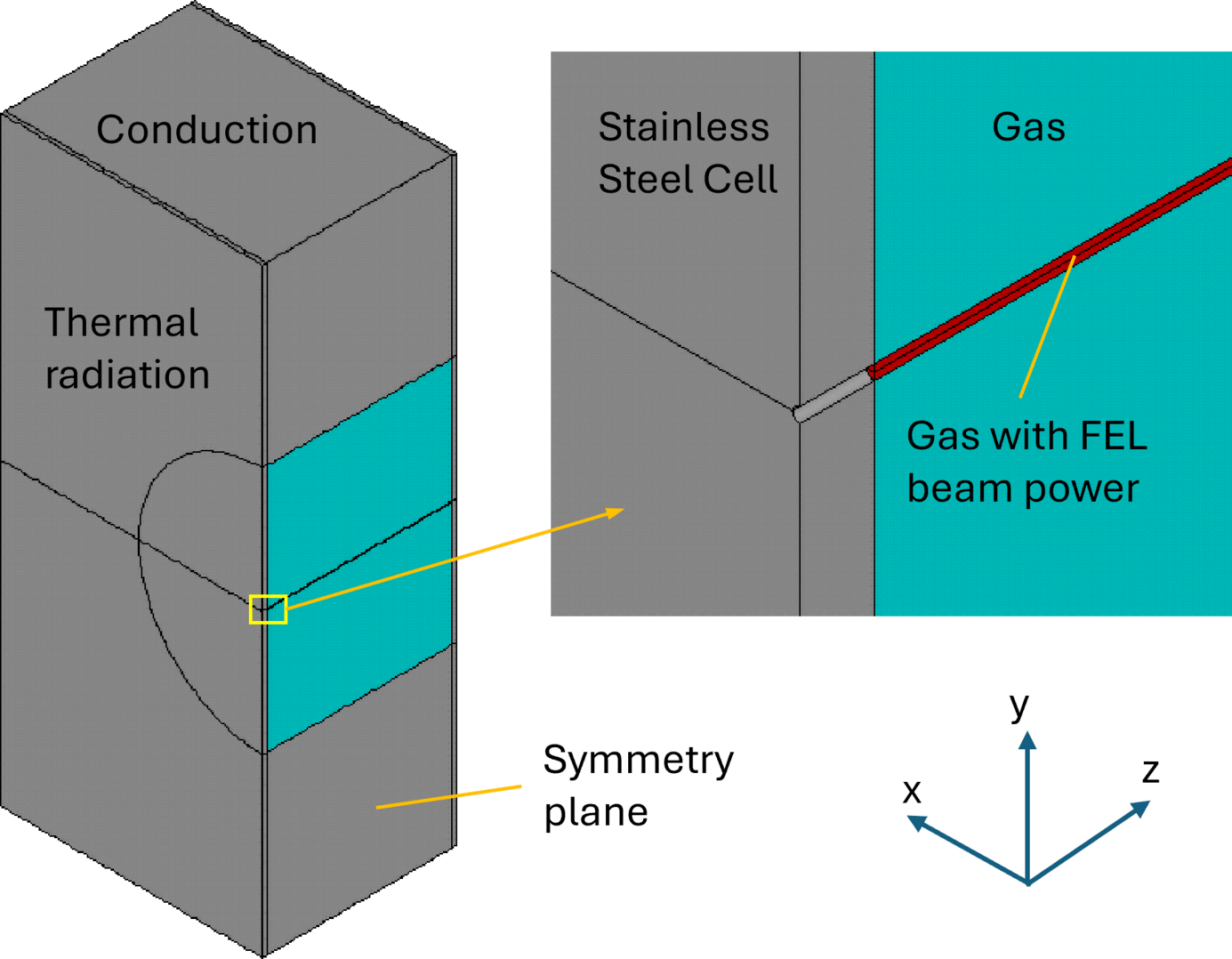
Finite-element model of half gas cell. The gas is shown in cyan, the stainless steel cell in gray and interaction volume of the gas with FEL beam in red. The origin of the coordinate system is at the center of the entrance section of the FEL beam.

We can use commercial FEA software (for instance, *ANSYS*) to simulate the gas power absorption and temperature distribution. Half of the gas cell is modeled as shown in Fig. 6 ▸, neglecting gas flow and natural convection. In this FEA model, the gas volume (cyan color) is a cylinder of 6 mm in diameter and 6.6 mm in length. The stainless steel cell (gray color) in the model is 14.5 mm in height, 12.7 mm in width, and 6.8 mm in length including 0.1 mm-thick entrance and exit walls. The interaction volume of the gas with FEL beam (red color) is a cylinder of diameter *D*_beam_ = 20 µm, and 6.6 mm in length.

### 4.2. Material properties

**Figure 7:**
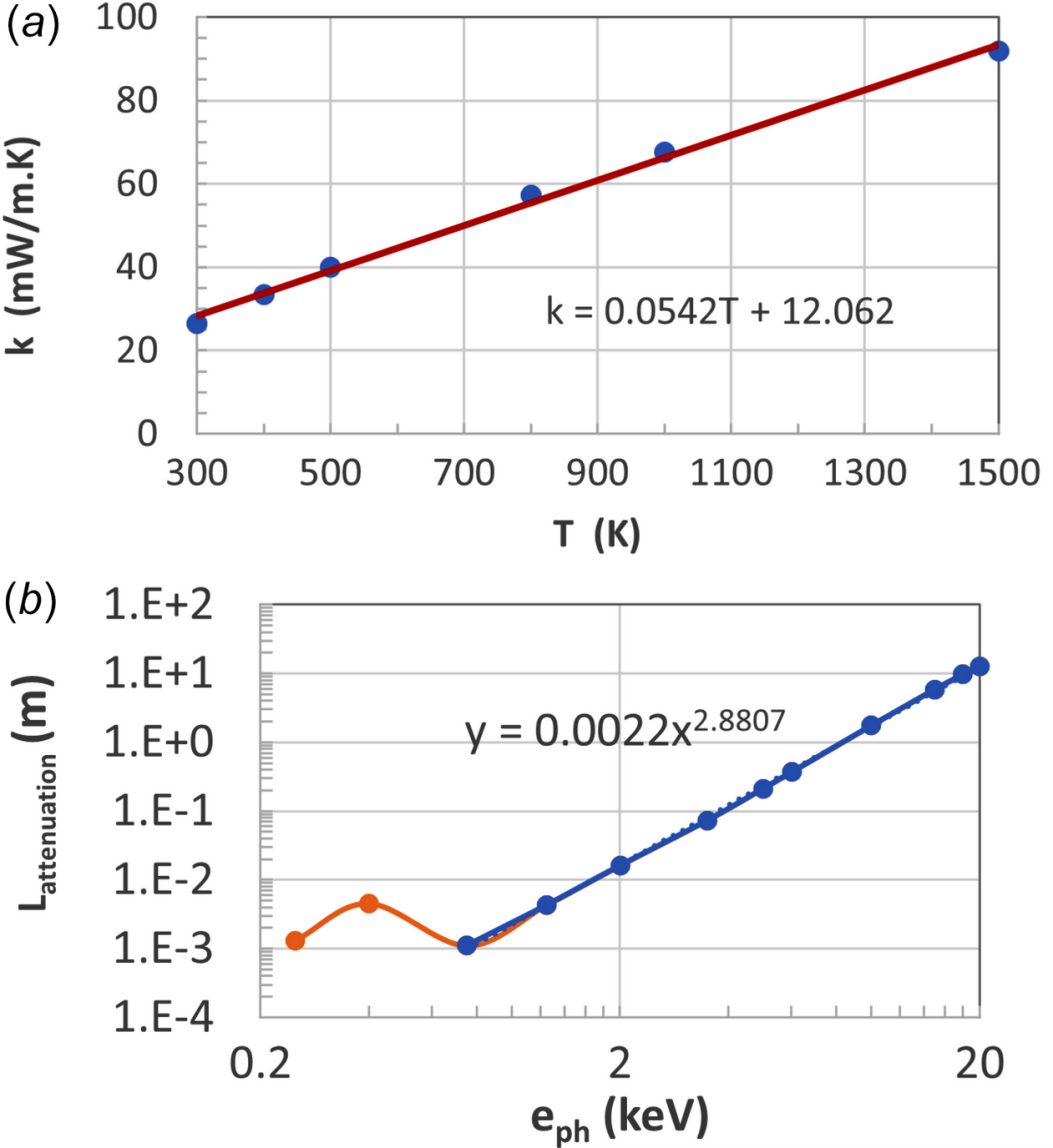
(*a*) Air thermal conductivity at 1 atm and (*b*) air attenuation length at room temperature and 1 atm.

The temperature of the air in the cell can vary over a large range. Therefore, the temperature-dependent material properties for air have been considered. The temperature-dependent thermal conductivity *k* (The Engineering Toolbox, 2009 ▸), and photon-energy-dependent attenuation length *L*_att_ of the air (https://henke.lbl.gov/optical_constants/) are given in Figs. 7 ▸(*a*) and 7(*b*), respectively. The absorption coefficient μ can be calculated as μ = 1/*L*_att_. Using the ideal gas law for the relationship between gas density ρ and temperature *T* at constant pressure, ρ*T* = ρ_0_*T*_0_, we can write the temperature-dependent absorption coefficient at any density or temperature as
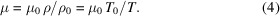
The temperature of the gas cell wall should be much closer to room temperature; therefore, constant material properties of stainless steel will be used here. Table 2 ▸ summarizes the material properties of stainless steel and air at standard conditions (1 atm and 20°C). The specific heat of air varies by less than 10% in the temperature range from 300 to 1800 K. We use the value of the specific heat at room temperature as shown in Table 2 ▸.

### 4.3. Boundary conditions for FEA

We assume that the FEL beam surface power density distribution on the cross section *Pa*(*x*, *y*) has a Gaussian shape,
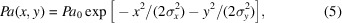
where *Pa*_0_, σ_*x*_ and σ_*y*_ are, respectively, the peak power density (W mm^−2^) at the center of the FEL beam and the standard deviation (mm) in the *x* and *y* directions. See Fig. 6 ▸ for the coordinate system used.

Combining equations (5) and (4), we have the volumetric power absorption density *Pv*(*x*, *y*, *z*) (W cm^3^) by gas,
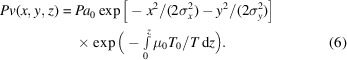
Here, μ_0_ is the absorption coefficient of the gas at a given photon energy *e*_ph_ and at room temperature *T*_0_. When the temperature of the gas increases, the density of the gas decreases, and the power absorption by the gas decreases. On the other hand, to calculate the temperature distribution of the gas, we need the power absorption of the gas. To solve this interdependent problem, we introduced an iterative algorithm in the FEA. A good estimate of the initial uniform temperature of the gas can improve the convergence of the iterative FEA simulation.

For a beam size of 8 µm (FWHM), the diameter of the interaction volume of the gas covers about 2.5×FWHM. The filling tube is not modeled in the FEA since the cross section of the tube is much smaller than the top surface area of the gas cell model. At the front and back surfaces, gas leaks from the two holes drilled by the beam of ∼20 µm diameter, as estimated from previous observations. The quantity of leaked gas can be estimated by using various tools as 0.308 to 0.452 atm L h^−1^. The time to recycle the air in the whole cell is 1.4 to 2 s. The thermal diffusion time over the radius of the gas cell (3 mm) is 0.036 s if the temperature of the air is 1000°C. The thermal diffusion time is much shorter than the time to fully recycle the gas in the cell. Therefore, the assumption of a temperature distribution in the gas undisturbed by gas flow is a reasonable approximation. Including the gas flow would lower the temperature and the transmitted power.

Gas in the cell partially absorbs the FEL beam power and is heated up in the interaction volume. To simplify the model, the heat is assumed to be transferred from the air in the center to the stainless steel walls of the cell by thermal conduction. Then the gas cell is cooled down by thermal conduction through the top surface, and marginally by thermal radiation on all lateral and bottom surfaces. The power from the leaked gas from the two holes on the front and back surfaces of the cell can be also estimated. The power balance in the gas cell can be expressed as
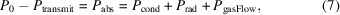
where *P*_0_ and *P*_transmit_ are the incident and transmitted FEL power. The absorbed power *P*_abs_ by the gas is the integral of the volumetric FEL power absorption *Pv*(*x*, *y*, *z*) over the whole volume *V* of the gas cell,
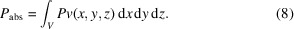
For the FEA simulation, we can use effective convection cooling for the heat power extraction from the gas through the gas cell walls as boundary conditions:

(1) On the top surface of the gas cell, the thermal conduction heat power can be expressed as *P*_cond_ = *A*_top_*h*_cond_ (*T* − *T*_0_) with *h*_cond_ = *k*/*l*_cond_. Here, *A*_top_, *T*, *T*_0_, *k*, *l*_cond_ are, respectively, the top surface area, the average temperature of that top surface, the ambient temperature, the thermal conductivity of stainless steel, and an effective conduction length above that top surface to the support of the gas cell reaching ambient temperature. If we use 10 mm as the value of *l*_cond_, we have *h*_cond_ = 0.0015 W mm^−2^ K^−1^.

(2) On all lateral and bottom surfaces, thermal radiation heat power can be approximated as *P*_rad_ = *A*_rad_ɛσ(*T*^4^ − *T*_0_^4^) = *A*_rad_*h*_rad_(*T* − *T*_0_), where *A*_rad_, ɛ and σ are, respectively, the total surface area of those surfaces, the thermal emissivity of stainless steel and the Stefan–Boltzmann constant. When the temperature of the gas cell wall is not too high, we can approximately have *h*_rad_ ≃ 4ɛσ*T*_0_^3^ = 3.7 × 10^−6^ W mm^−2^ K^−1^.

(3) On the front and back surface of the FEL beam and gas interaction volume, the power loss due to the leaked gas can be expressed as *P*_gasFlow_ = 2*A*_hole_*h*_cv-leak_(*T* − *T*_0_) = ρ*c*_p_(*T*_*a*1_*q*_*a*1_ + *T*_*a*2_*q*_*a*2_ − *T*_*a*0_*q*_*a*0_), where *A*_hole_, ρ, *c*_p_, *T*_*ai*_, *q*_*ai*_ and *q*_*a*0_ are, respectively, the surface area of the hole, the density of the gas, the specific heat of the gas, the temperature and flow rate of leaked gas on hole *i*, and the flow rate of the gas. By assuming the same temperature and flow rate of leaked gas through both holes, and considering mass conservation and flow rate *q*_*a*_ = *vA*_hole_ (where *v* is the leaked gas speed), we have*h*_cv-leak_ =*v*ρ*c*_p_ = *v*ρ_0_*c*_p_*T*_0_/*T* = (0.17–0.25)*T*_0_/*T* W mm^−2^ K^−1^.

Equation (6) is used to model the volumetric power absorption by the gas, and an estimate of a uniform gas temperature of *T*_ini_ = 1000 + 200*P*_FEL_ (K) is used for the first iteration of FEA. This choice of *T*_ini_ improves very significantly the convergence of the iterative simulations. Then in iteration *i*, we use the temperature distribution results from iteration *i* − 1 to calculate the volumetric power density distribution using equation (6), and the total absorbed power by the gas *P*_abs_(*i* − 1) using equation (8). We iterate the FEA until the following convergence criterium is satisfied,
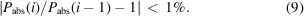


### 4.4. Simulation results

We have performed FEA for FEL powers *P*_FEL_ between 0.2 and 18 W, beam sizes of 4 and 8 µm FWHM, gas cell lengths of 4.5, 6 and 6.6 mm, and gas pressures of 0.1, 0.5 and 1 atm. The results of these simulations were used to determine the optimal setup for the experiments: 1 atm air in a 6.6 mm-long gas cell. The difference in absorbed power with the beam size changed from 4 to 8 µm is +4% for *P*_FEL_ = 1 W and +1% for *P*_FEL_ = 10 W. As the computation time with a 4 µm beam diameter is much longer than with an 8 µm beam diameter, the 8 µm beam size was used primarily in the simulations. FEA results also show that the power loss due to the leaked gas is less than 0.6%.

**Figure 8:**
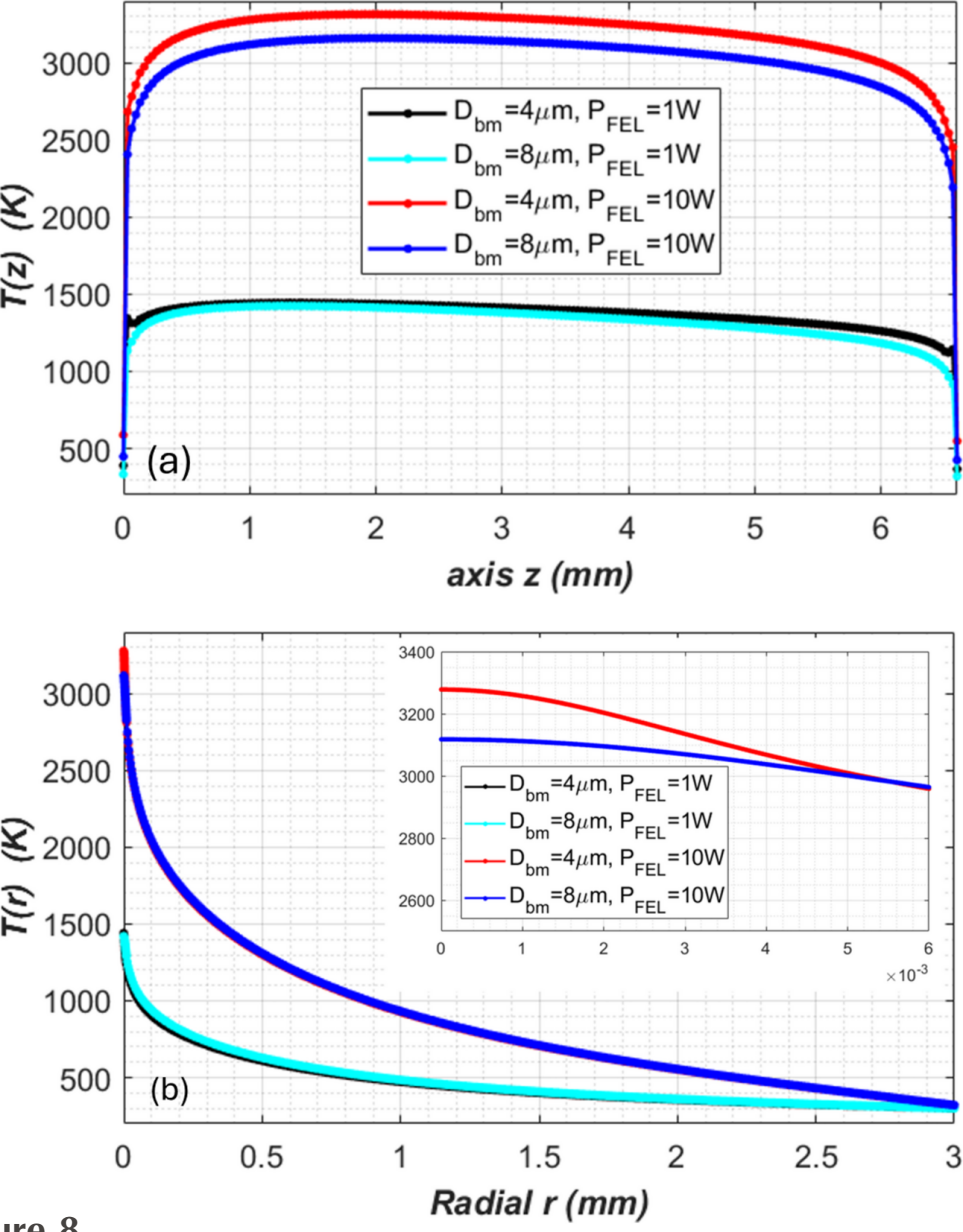
Temperature profile of the air (*a*) along the *z* axis at the center of the gas cell and (*b*) along the radial direction for 1 and 10 W input FEL power and 4 and 8 µm beam size.

The temperature distribution of the air along the center of the cell (*z*) and in the radial direction (*r*) is plotted in Fig. 8 ▸ for 1 and 10 W input FEL power, and for 4 and 8 µm beam size. Fig. 8 ▸ shows that the temperature of the air on the center of beam path is quite flat with slight decrease towards the end. The high temperature gradient of the air at the most upstream and downstream ends is a consequence of the cooling effects from the upstream and downstream cell walls. Fig. 8 ▸(*b*) shows that in the radial direction of the gas cell the temperature gradient extends far beyond the X-ray beam diameter. When reducing the beam diameter from 8 to 4 µm, the incident power density increases leading to a higher temperature of the gas. But the higher temperature, in turn, results in lower gas density and reduced power absorption. The combined effects of reducing the beam size on the temperature distribution are significantly attenuated. The extended radial temperature gradient provides an explanation for the simulations’ insensitivity to the beam size, 4 or 8 µm. As explained in Section 4.1, the FEA results overestimate the actual transmission by about 70% and should therefore be considered as an upper bound.

**Figure 9:**
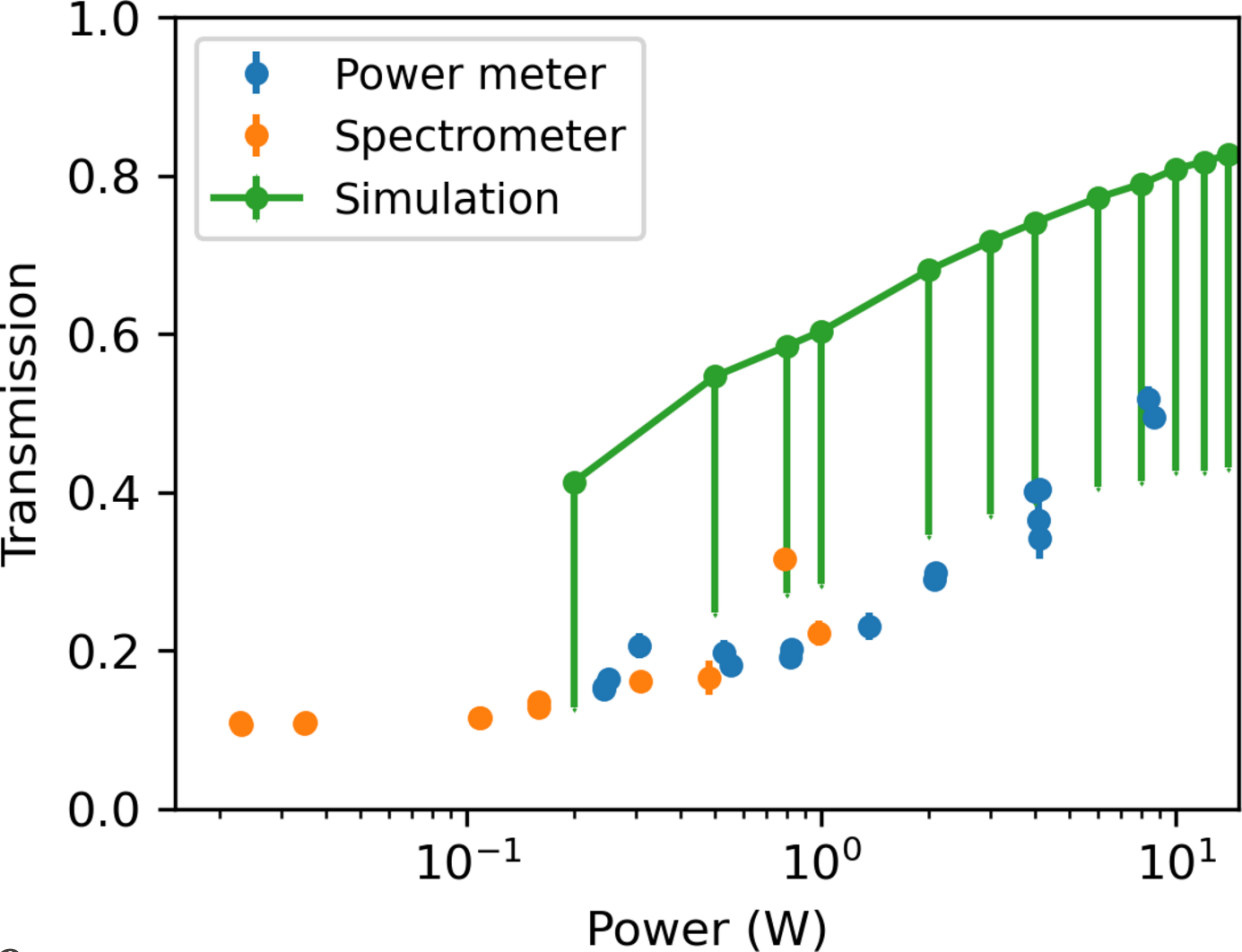
The calculated air transmission through the gas cell for a beam size of 8 µm FWHM together with the measured transmission.

Fig. 9 ▸ shows the calculated air transmission together with the measured transmission. As described in Section 4.1, the calculated results are based on an approximate, but conservative, model used for the design of radiation safety systems (Zhang, 2022 ▸). Shown by the vertical bars in Fig. 9 ▸, the uncertainties, resulting in lower transmission values, are estimated based on the discussion in Section 4.1. For 10 W FEL power absorption by gas cell, we assume that *f*_nt_ = 30% of FEL power is lost by non-thermal processes (plasma and photon electrons, secondary photon emission, *etc.*). The power evacuated by thermal conduction simulated here *P*_abs_ is *f*_co_ = 30% under-estimated due to the convection heat transfer neglected. Therefore, the effective transmitted FEL power*P*_tr-eff_ is then
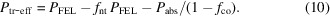
In comparison with the measurements, the calculated transmission is higher, as anticipated. This result confirms that the air attenuation calculations are conservative up to ∼10 W and appropriate for use in radiation physics calculations at this power level. The results also suggest that it is safe to ramp up power to the next bootstrap power level at which point air attenuation among other bootstrap tests can be re-performed.

## 5. Conclusions and summary

X-ray absorption measurements have been performed on air enclosed in a gas cell. The air transmission was observed to increase with increasing X-ray power demonstrating a tunneling effect. The measurements have been compared with simulations based on iterative calculations of the air temperature and density. The simulations were shown to be conservative up to ∼10 W of FEL, predicting higher air transmission than the measured result. In the future, air attenuation measurements will be conducted at higher photon energies and photon powers produced by the LCLS-II-HE facility.

## Supplementary Material

Video S1 - video of Fig. 2. DOI: 10.1107/S1600577526004911/ing5022sup1.gif

## Figures and Tables

**Table 1:** Measurement parameters

Photon energy	316, 346 eV
FEL pulse energy	∼340 µJ
Pulse energy at cell	∼250 µJ
Repetition rates	100–33.2 kHz
Attenuator transmission	0.07–1
Power	0.02–8.6 W (at cell)
Focus	2–10 µm
Gas	Air
Pressure	760 torr
Cell length	6.6 mm

**Table 2:** Material properties of stainless steel and air at standard conditions (1 atm and 20°C)

	Stainless steel	Air (1 atm, 20°C)
Thermal conductivity, *k* (W mm^−1^ K^−1^)	0.015	2.58 × 10^−5^
Heat capacity, *c*_p_ (J kg^−1^ K^−1^)	450	1041
Density, ρ (kg mm^−3^)	7.86 × 10^−6^	1.15 × 10^−9^
Attenuation length at 350 eV (mm)		3.045
Thermal emissivity	0.6	

## Data Availability

The data that support the findings of this study are available from the authors upon reasonable request.
