# Supplementary figures and images for: Air attenuation of high power XFEL beams
Source: J Synchrotron Radiat. 2026 Jun 5;33(Pt 4):913–20. doi: 10.1107/S1600577526004911 (PMC13344588; doi:10.1107/S1600577526004911)

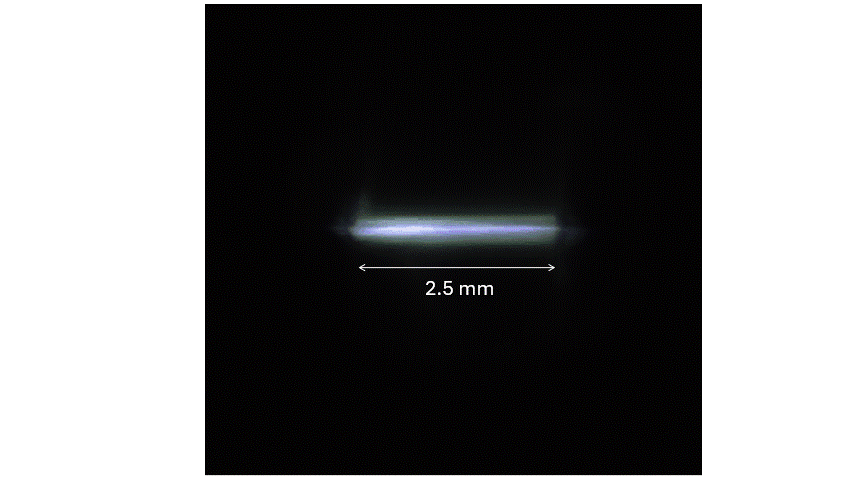

Supplement: Supplementary file 1 [file s-33-00913-sup1.gif]
